# Circulating long non-coding RNAs in cancer: current status and future perspectives

**DOI:** 10.1186/s12943-016-0524-4

**Published:** 2016-05-17

**Authors:** Peng Qi, Xiao-yan Zhou, Xiang Du

**Affiliations:** Department of Pathology, Fudan University Shanghai Cancer Center, Shanghai, 200032 China; Department of Oncology, Shanghai Medical College, Fudan University, Shanghai, 200032 China; Institute of Pathology, Fudan University, Shanghai, 200032 China; Institutes of Biomedical Sciences, Fudan University, Shanghai, 200032 China

**Keywords:** Biomarker, Cancer diagnosis, Cancer prognosis, Sensitivity, Specificity, Circulating lncRNAs, Exosomes

## Abstract

Long non-coding RNAs (lncRNAs) comprise a diverse class of RNA transcripts >200 nucleotides in length with limited protein-coding potential. In addition to their possible role in cancer biology, circulating lncRNAs have emerged as a new class of promising cancer biomarkers, with independent studies demonstrating the feasibility of their use as tools in the diagnosis and prognosis of different types of malignancies and for predicting and possibly monitoring treatment response. However, critical issues are represented by nonuniform sample choice, handling and processing, blood cell contamination during sample preparation and the lack of consensus regarding data normalization. In this review, we discuss the value of circulating lncRNAs in the clinical setting, particularly with respect to their possible implementation as diagnostic and prognostic markers in cancer. Although the great potential of circulating lncRNAs as cancer biomarkers would be an important development in disease management, both intrinsic and extrinsic factors that may affect their measurement have not been fully characterized. Moreover, the clinical significance of circulating lncRNA may not be proven without a global consensus regarding procedures and standardized protocols for their detection.

## Background

Contrary to the original expectation that higher mammals would have a greater number of genes, it is now clear that the number of protein-coding genes is similar between humans and mice [1]. In fact, the complexity of a species is more closely associated with the number of non-coding RNAs (ncRNAs) than with the number of protein-coding genes [2], and the role of such ncRNAs in modulating gene expression has long been recognized. To date, various classes of ncRNAs with different targets and functions have been identified [3, 4], and these molecules can be grouped into two major classes: small non-coding RNAs, including microRNAs (miRNAs), which are perhaps the best described [5]; and long non-coding RNAs (lncRNAs), which were only recently discovered [6, 7]. A given lncRNA transcript may be further classified into different categories depending on the criteria applied [8].

lncRNAs are classically defined as RNA transcripts greater than 200 nucleotides in length that have no or limited protein-coding potential. According to the GENCODE analysis (available at www.gencodegenes.org) of the last version of the Ensembl human genome annotation (GRch38, version 23 from March 2015; [9]), 27,817 transcripts originating from 15,931 genes can be identified as lncRNAs [10]. Basal expression of lncRNAs in many tissues has been shown to be important for various homeostatic processes, including gene imprinting [11] and cell differentiation and organogenesis [12]. There is also a strong association between deregulated lncRNA expression and the development of disease. Indeed, lncRNAs have been linked to the modulation of oncogenic and tumor-suppressing pathways [13–17], and lncRNA signatures from normal cancer tissues and metastases have been used to classify different types of cancer, representing potential biomarkers for diagnosis, prognosis, and therapy [18–21]. In addition, although the precise mechanism of lncRNA release into the extracellular environment is not completely understood, recent studies have suggested that some lncRNAs are also present in serum, plasma and other bodily fluids in a stable form protected from endogenous RNases. Therefore, circulating lncRNA levels are well suited for noninvasive analysis of patient samples [22, 23]. In this review, we focus on studies and technical issues concerning the evaluation of circulating lncRNAs and their potential as biomarkers for cancer.

## Deregulated circulating lncRNAs in different cancers

Cancer is a leading cause of death worldwide, and largely due to the lack of early detection methods, many cancers are diagnosed at advanced stages with poor prognosis [24]. Thus, to reduce cancer-related mortality, it is crucial to develop specific and sensitive biomarkers for identifying cancer in early stages. Because most of the existing oncology markers are based on protein targets, proteins have been the traditional focus of cancer-biomarker discovery; however, little progress has been made despite substantial efforts over the past few decades. Circulating nucleic acids (CNAs) are extracellular nucleic acids found in cell-free serum, plasma and other bodily fluids of healthy subjects and cancer patients. We and other researchers have demonstrated that human serum or plasma contains miRNAs that are significantly upregulated or downregulated in various types of cancer and are thereby of high diagnostic value for screening [25–28]. Following the growing interest in miRNAs as diagnostic and prognostic biomarkers, attention is now shifting to lncRNAs and their diverse functions. Indeed, over the last few years, there has been increasing importance given to circulating lncRNAs as biomarkers for early cancer diagnosis, disease evolution and poor prognosis.

### Prostate cancer (PCa)

PCa is the second most common cancer and the sixth leading cause of cancer-related death among men worldwide. Currently, diagnostics rely on the serum detection of the androgen-regulated serine protease PSA, the expression of which is highly specific to prostate tissue. Unfortunately, the routine use of PSA as a diagnostic marker is complicated by several issues; specifically, elevated levels of serum PSA are not specific to PCa because they are also associated with benign prostatic hyperplasia and prostatitis, and the early detection of PCa using PSA and biopsy is associated with overdiagnosis and overtreatment of clinically significant PCa [29].

PCA3 (Prostate Cancer Antigen 3 lncRNA; also referred to as DD3), a well-investigated lncRNA in PCa, was found to be strongly overexpressed in more than 95 % of primary PCa specimens and metastasis [30]. As it is not expressed in other normal human tissues, PCA3 is the most PCa-specific gene known to date. PCA3 can be identified in urine, and the PROGENSA PCA3 test is the first urine-based molecular diagnostic test approved by the Food and Drug Administration [31]. Furthermore, the use of PCA3 as a biomarker in clinical practice has been extensively reviewed. A meta-analysis of several studies confirmed the validity of urine PCA3 levels for prostate cancer diagnosis, with a summary sensitivity of 62 % and a specificity of 75 %. In receiver operating characteristic curve (ROC) analysis, this result translated into an area under the ROC curve (AUC) of 0.75, further supporting PCA3 as a reasonable marker for prostate cancer diagnosis [32]. Similar results were obtained in a second independent meta-analysis [33] in which the sensitivity and specificity for prostate cancer diagnosis were 57 % and 71 %, respectively, and the AUC was 0.7118 [33]. As its levels correlate with tumor aggressiveness as classified by the Gleason score, circulating PCA3 can also reflect the aggressiveness of prostate cancer [34].

In addition to PCA3, several novel PCa-specific or PCa-associated lncRNAs are on imminent, but none have proven to be usable in a clinical test for PCa. One of the most-studied lncRNAs, MALAT-1 (Metastasis-Associated Lung Adenocarcinoma Transcript 1), is overexpressed during prostate cancer progression [35]. In early studies, fragments from different regions of the MALAT-1 transcript were detected at higher copy numbers in the plasma of patients with prostate cancer than in non-prostate cancer patients. With a sensitivity and specificity of 58.6 % and 84.8 %, respectively, MALAT-1 has been proposed as a biomarker for prostate cancer diagnosis [36]. Although the sensitivity of plasma MALAT-1 was found to be lower than that for serum PSA, Wang et al. later reported that the MALAT-1 model would prevent approximately 30.2–46.5 % of unnecessary biopsies in patients with serum PSA levels of 4–10 ng/mL [37]. These data indicate that MALAT-1 is a promising biomarker for prostate cancer detection. lncRNA-PCAT-18 (Prostate Cancer-Associated Noncoding RNA Transcript 18), which was recently discovered by RNA sequencing, also exhibits a highly specific expression pattern in prostate cancer: the gene is specifically expressed in prostate tissue and is upregulated in prostate cancer compared to other benign and malignant tissues. Similar to the aforementioned lncRNAs, PCAT-18 can be detected in plasma, and its expression increases incrementally as prostate cancer progresses from localized to metastatic disease, suggesting that PCAT-18 is a potential biomarker for metastatic prostate cancer [38].

### Hepatocellular carcinoma (HCC)

Although numerous lncRNAs are misregulated in a variety of human cancers, few have been associated with a single type of cancer. For example, HULC (Highly Upregulated in Liver Cancer) is expressed at high levels in HCC and colorectal carcinomas that have metastasized to the liver [39, 40], but not in primary colon tumors or non-liver metastases, with increased plasma HULC levels observed in 63 % of HCC patients [41]. Unfortunately, no data are available regarding the specificity and sensitivity of circulating HULC for the diagnosis of HCC patients. Furthermore, Li et al. demonstrated that circulating HULC and LINC00152 were significantly upregulated in HCC patient plasma in training and validation sets [42]. The AUCs of the two validated lncRNA signatures were 0.78 and 0.85, respectively. Additionally, the combination of HULC and LINC00152 showed a moderate ability to discriminate between HCC and a control, with an AUC of 0.87, whereas the AUC in combination with AFP was 0.89 and showed a positive correlation with tissue expression.

Other less commonly studied lncRNAs have recently been proposed for HCC diagnosis. Tang et al. identified upregulation of RP11-160H22.5, XLOC_014172 and LOC149086 transcripts in the plasma of HCC patients compared to cancer-free controls, and combination of the three lncRNAs yielded better scores for HCC diagnosis compared to each individual lncRNA, with a merged AUC of 0.896, a sensitivity of 82 % and a specificity of 73 % [43]. Interestingly, lncRNAs XLOC_014172 and LOC149086 also have prognostic value for metastasis prediction; these lncRNAs were able to distinguish HCC patients with metastasis from patients without, showing a sensitivity and specificity of 91 % and 90 %, respectively (AUC for the combined lncRNAs of 0.934) [43]. Another study identified lncRNA-AF085935 in serum as a potential biomarker for HCC diagnosis, distinguishing not only HCC patients from healthy control individuals but also HCC patients from hepatitis B-infected patients, with AUCs of 0.96 and of 0.86, respectively [44].

### Gastric cancer (GC)

H19 is reported to be upregulated in GC tissues compared to paired noncancerous tissues. Furthermore, H19 overexpression promoted the GC characteristics of proliferation, migration, invasion and metastasis by directly upregulating ISM1 and indirectly suppressing CALN1 expression via miR-675 [45]. Independent reports reveal consistent results using plasma H19 for GC diagnosis. Arita et al. [46] found that the level of plasma H19 was significantly higher in GC patients than in healthy controls, with a sensitivity of 74 %, a specificity of 58 % and an AUC of 0.64 for GC diagnosis, and Zhou et al. obtained a sensitivity of 82.9 %, a specificity of 72.9 % and an AUC of 0.838 [47]. Furthermore, H19 expression enabled differentiation between early-stage GC and controls, with an AUC of 0.877, a sensitivity of 85.5 % and a specificity of 80.1 % [47]. Moreover, the increase in H19 in patient plasma in both studies was significantly reduced in postoperative samples, suggesting a direct correlation between cancer tissue mass and circulating H19 concentrations.

Some lncRNAs show similar trends in different cancer types, as reported in different studies. For example, increases in LINC00152 concentrations in HCC patient plasma samples [42] were also observed in GC patients [48, 49], and plasma LINC00152 is a promising noninvasive biomarker for GC screening, with an AUC of 0.657, a sensitivity of 48.1 % and a specificity of 85.2 %. Li et al. [49] also analyzed the levels of LINC00152 in plasma and plasma-derived exosomes from GC patients and found no statistically significant difference in expression between the two sample types, suggesting that at least the majority of plasma LINC00152 is derived from exosomes. Nonetheless, as LINC00152 can function as an oncogene to promote tumorigenesis via different signaling pathways in GC and HCC [50–52], the relevance of circulating LINC00152 in different cancer types should be investigated further.

Thus far, plasma/serum and urine are the bodily fluids most commonly used for lncRNA profiling in cancer patients, though other fluids have also been tested. For instance, LINC00152 levels were found to be significantly increased in gastric juice from 17 GC patients compared to 16 healthy controls [48]. Reports have also reported significantly higher levels of lncRNA AA174084 in gastric juice from GC patients than healthy individuals as well as patients with other gastric mucosa lesions, corresponding to an AUC of 0.848 for gastric cancer diagnosis [53]. AA174084 in digestive fluids is a robust biomarker, diagnosing disease with a sensitivity of only 0.46 but a specificity of 0.93, whereas its plasma levels could not be used to distinguish GC patients from healthy individuals. Urothelial Carcinoma-Associated 1 (UCA1) is another lncRNA detected in gastric juice, the aberrant expression of which is always accompanied by a broad range of human cancers [54], with Zheng et al. reporting that gastric juice from GC patients had significantly higher levels of UCA1 than gastric juice from normal subjects [55].

In addition to diagnostic applications, circulating lncRNAs also have prognostic value for GC patients. For instance, Liu et al. examined the level of plasma Fer-1-Like Protein 4 (FER1L4), a newly identified lncRNA that is downregulated in GC tissues, and observed no differences between preoperative GC patients and healthy individuals; however, a sharp decline in expression was observed in GC patients two weeks after surgery [56].

Overall, the sensitivity and specificity of any single tumor-associated circulating lncRNA as a biomarker remain poor; however, a circulating lncRNA signature, especially the combination of various circulating lncRNAs, can considerably promote the efficiency of GC detection. Indeed, we measured the expression levels of 39 candidate cancer-associated circulating lncRNAs by RT-qPCR in the sera of 110 patients with GC, 15 patients with benign gastric ulcer and 106 healthy individuals [57]. Our results showed that the combination of CUDR, LSINCT-5 and PTENP1 provided the greatest ability to distinguish GC patients from healthy controls, with an AUC of 0.92, a sensitivity of 74.1 % and a specificity of 100 %. In addition, this three-lncRNA signature suggested a strong diagnostic value, with an AUC of 0.832, a sensitivity of 77.8 % and a specificity of 97.0 % for early GC detection, and was sufficiently sensitive and specific for distinguishing benign peptic ulcers from GC, with an AUC of 0.902, a sensitivity of 91.7 % and a specificity of 83.3 %. These data support this serum three-lncRNA signature as a novel biomarker candidate for GC detection.

### Bladder cancer

UCA1 has been found to be highly expressed in bladder cancer tissues compared to adjacent normal tissues [58]. Interestingly, circulating UCA1, including blood and urine, can also be detected in bladder cancer patients [58, 59]. Importantly, this marker allows for distinguishing bladder cancer from other diseases related to the urinary tract, such as neurogenic bladder, renal cell carcinoma, and upper urinary tract restriction or reflux, with an overall specificity of 91.8 %. An ROC analysis of UCA1 detection yielded an AUC of 0.882, suggesting reasonable efficacy of this lncRNA in bladder/urothelial cancer diagnosis [58]. Srivastava et al. obtained a similar result, with even higher urine transcript levels detected as the tumor stage progressed [60]. Furthermore, circulating lncRNAs might represent not only promising noninvasive diagnostic and prognostic tools but also a means for predicting and monitoring the efficacy of anticancer treatments. For example, UCA1 is upregulated in the blood of patients with advanced bladder cancer after cisplatin-based combination chemotherapy, suggesting that UCA1 may be used to predict the outcome of chemotherapy for bladder cancer [61]. Taken together, these limited clinical data indicate that circulating UCA1 is a promising biomarker for bladder cancer diagnosis and therapeutic monitoring. This promising lncRNA has also been detected in serum, with significantly higher levels in the serum of patients with HCC than in that from patients with chronic HCV infection or healthy volunteers. Higher expression of serum UCA1 has also been associated with advanced clinical parameters in HCC [62]. Importantly, tissue levels of UCA1 are strongly correlated with levels in serum.

### Cervical cancer

Only one study has explored the diagnostic potential of changes in concentrations of specific circulating lncRNAs in cervical cancer. This low number of cervical cancer studies is likely due to the fact that most localized cervical cancer tissues are accessible and can be diagnosed by regular cytopathological examination. HOX Transcript Antisense RNA (HOTAIR) is a well-studied lncRNA that interacts with Polycomb Repressive Complex 2 (PRC2) and trimethylated H3K27 at the HOXD gene locus [63]. Huang et al. reported that elevated HOTAIR levels were significantly associated with tumor progression and metastasis in cervical cancers [64], and Li et al. subsequently demonstrated that HOTAIR expression was significantly upregulated in the serum of cervical cancer patients [65]. Additionally, increased levels of HOTAIR were associated with advanced tumor stages, adenocarcinoma, lymphatic vascular-space invasion, and lymphatic-node metastasis, and high serum levels of HOTAIR were significantly correlated with tumor recurrence and shorter overall survival.

### Colorectal cancer (CRC)

CRC is one of the leading causes of cancer-related death worldwide. Because a significant proportion of patients who develop CRC have no specific risk factor for the disease, the best indicator of prognosis is the stage at diagnosis. Although several screening tests, including fecal occult-blood testing, colonoscopy and stool DNA testing, have been available for years, none of these methods have been established as a well-accepted screening tool due to their low adherence rates and low sensitivity and reproducibility. Using microarray analysis and multi-stage validation by RT-qPCR, Shi et al. found a panel of three lncRNAs (XLOC_006844, LOC152578 and XLOC_000303) to be significantly upregulated in CRC plasma samples compared to those from healthy controls [66]. This three-lncRNA signature has a strong diagnostic value, with an AUC of 0.919 in the training set and an AUC of 0.975 in the validation set. Furthermore, these three lncRNAs are sufficiently stable in human plasma.

Another study by Yue et al. examined 150 blood samples, including 50 from preoperative colon cancer patients, 50 from those patients one month after surgery and 50 from healthy individuals. The authors found no difference in levels of circulating FER1L4 between preoperative patients and healthy persons but decreased levels in 70 % (35/50) of colon cancer patients one month after surgery [67].

### Non-small-cell lung cancer (NSCLC)

Lung cancer remains the leading cause of cancer mortality worldwide, with the major histological subtype, non-small-cell lung cancer (NSCLC), accounting for approximately 80–85 % of all cases. Unfortunately, most patients are diagnosed at advanced stages, with an overall survival rate of only 15 %. To date, biomarkers for NSCLC include tumor-liberated proteins, such as CEA, NSE, TPA, chromogranin, CA125, CA19-9 and CYFRA21-1 [68, 69]; however, despite their high sensitivity, all of the biomarkers currently applied clinically have low specificity [70]. Hu et al. measured 21 NSCLC-related lncRNAs in plasma samples from NSCLC patients and healthy volunteers [71] and reported were significantly increased circulating SPRY4-IT1, ANRIL and NEAT1 in the former in both training and validation sets. ROC analysis revealed that plasma ANRIL provided the highest diagnostic performance, with an AUC of 0.798, and further combination with SPRY4-IT1, ANRIL and NEAT1 yielded higher power, with a sensitivity of 82.8 %, a specificity of 92.3 % and an AUC of 0.876. These results indicate that circulating SPRY4-IT1, ANRIL, and NEAT1 might serve as signatures for predicting NSCLC. As these three lncRNAs are correlated with tumor size, high circulating levels may indicate a higher tumor burden during NSCLC progression.

### Esophageal squamous cell carcinoma (ESCC)

ESCC is one of the most fatal malignancies in humans; patients tend to present with dysphagia at an advanced stage, and the five-year survival rate is less than 15 % [72]. Current biomarkers, such as serum Squamous Cell Carcinoma Antigen (SCCA), CA19-9, and CEA, are classic tumor markers used in the management of patients with ESCC. However, due to their insufficient diagnostic sensitivity and specificity, these tumor markers have limited utility in the early detection of ESCC [73]. Tong et al. measured 10 ESCC-related lncRNAs in plasma from ESCC patients and healthy volunteer donors [74] and found that the plasma levels of POU3F3, HNF1A-AS1 and SPRY4-IT1 were significantly higher in ESCC patients. According to ROC analysis, plasma POU3F3 provided the highest diagnostic performance for ESCC detection, with a sensitivity of 72.8 %, a specificity of 89.4 % and an AUC of 0.842. Moreover, the use of POU3F3 and SCCA in combination provided for a more effective diagnosis, with a sensitivity of 85.7 %, a specificity of 81.4 % and an AUC of 0.926. Most importantly, this combination effectively detects ESCC at an early stage (80.8 %).

### Breast cancer (BC)

BC is the second leading cause of cancer-related death among women worldwide, and delayed diagnosis, recurrence, and metastasis are the main obstacles for treating BC. Although blood-based testing is currently an ideal approach for detecting biomarkers in cancer due to its ease of use and low invasiveness [75], conventional serum biomarkers, such as CEA and CA125, are of limited clinical utility, and measurement of these biomarkers in BC patients remains controversial [76, 77]. By applying lncRNA-microarray analysis and subsequent RT-qPCR validation, Xu et al. found that lncRNA RP11-445H22.4 was highly expressed in BC tissues and serum from BC patients compared to controls [78]. Indeed, with a sensitivity of 92 %, a specificity of 74 % and an AUC of 0.904 (values that are better than those of other conventional biomarkers, including AFP, CEA, CA125 and CA153), serum RP11-445H22.4 may constitute a new potential biomarker for BC. Nonetheless, research concerning lncRNAs as biomarkers for BC is still in its infancy, and further investigations as well as large-scale validation studies are necessary before application in the clinical setting will be possible.

### Preanalytical and analytical variables affecting circulating lncRNA studies

Although several published papers have demonstrated the feasibility of utilizing circulating lncRNAs as putative cancer biomarkers, many preanalytical and analytical aspects, as well as donor-related factors, may interfere with the accurate quantification of circulating lncRNAs. Clearly, future studies will need to account for these factors.

### Sample choice/Starting material

The lncRNAs present in the circulation can be quantified using different sources (i.e., whole blood, plasma, serum, urine and gastric juice). Although the influence of blood cell lncRNAs (contained in red and white blood cells and platelets) on circulating lncRNA analysis has not been explored in detail, whole blood may not be a preferred biological fluid for detecting circulating RNAs, at least with regard to miRNAs [79]. The complete removal of cellular components and the identification of hemolyzed plasma/serum specimens that could impair accurate lncRNA quantification are mandatory even for studies involving plasma (the liquid part of blood, containing fibrinogen and collected in the presence of an anticoagulant) and serum (the liquid part of the blood obtained after clotting). Thus far, only one study has compared these two types of biological fluid in terms of the amount of circulating lncRNA [74], and univocal results have not been obtained because only three lncRNAs were analyzed. In fact, we observed higher lncRNA concentrations in serum compared to plasma due to the release of lncRNA from blood cells (such as platelets) during coagulation (unpublished data). Various separation methods for plasma and serum from whole blood and the choice of fresh or frozen fluids should also be explored in future studies. As lncRNAs are highly stable in the circulation, minimal or no differences have been found after repeated freeze-thaw cycles [57]. Nevertheless, unnecessary freeze-thawing should be avoided (even to a limited extent), as it may result in poorly represented lncRNA species being overlooked due to degradation. Moreover, plasma/serum samples from patients undergoing inflammatory processes could be contaminated with a high number of white blood cells [80], and exclusion of such patients may be advisable. Despite our above discussion of the technical issues known to be associated with blood collection and serum/plasma preparation, we cannot exclude the impact of other unknown factors on lncRNA profiling. Thus, standard operating procedures (SOPs) for blood collection and plasma/serum preparation are needed to minimize confounding effects.

### Extraction methods

Several extraction methods have been used in studies of circulating lncRNA. These methods can be divided into two different main categories: guanidine/phenol/chloroform-based protocols and commercial kits using columns. To date, no systemic results comparing commercial column-based kits with the TRIzol extraction method using different body fluids have been reported. In our experience, column-based methods perform better than TRIzol extraction due to the presence of organic and phenolic contaminants in TRIzol-extracted RNA (unpublished data), which is common when TRIzol is used to extract RNA from body fluids. In contrast, no consistent results have been obtained regarding differences in column-based methods, suggesting that comparisons of different extraction methods and standardization are still needed.

Another note is warranted regarding circulating RNA quantification. Regardless of the specific lncRNA detection method used, many research groups have quantified circulating RNA using a NanoDrop spectrophotometer before analyzing the sample for lncRNA content. However, RNA extracted from plasma/serum may be undetectable using this instrument. In addition, many diseases, including cancer, may cause the release of nucleic acids into the circulation, leading to a significantly higher level of circulating RNA in cancer patients than in healthy subjects. Thus, it may be more accurate to use an equal volume of input rather than equal amounts of RNA when analyzing circulating biomarkers.

### Techniques and obstacles in lncRNA measurement

The accurate measurement of circulating lncRNA poses several challenges, such as the low abundance of these nucleic acids in body fluids, normalization and data analysis. Nonetheless, several methods for measuring from one to thousands of lncRNAs have been applied.

RT-qPCR is a well-established method used to detect and quantify circulating lncRNAs; however, the cost per sample is high, and the throughput is low. Assays allowing for the measurement of a panel of lncRNAs, such as the Human Disease-Related LncRNA Profiler by System Biosciences SBI, have recently been developed. The data generated from this type of analysis are in terms of CT and thus do not require further bioinformatic manipulation. However, such technology also presents some disadvantages, such as the possibility of detecting only annotated lncRNAs and the fact that only a medium throughput can be attained.

Several commercial lncRNA microarray platforms (e.g., Human LncRNA Array by Agilent) are available and can be used to measure circulating lncRNAs [66]. The advantage of the microarray method is its ability to assess up to thousands of lncRNAs in one assay and to provide a large number of candidate biomarkers for diagnostic purposes in cancer; however, only previously described biomarkers already in the lncRNA-related database can be detected. This methodology has a high throughput and is less expensive than amplification-based arrays, but it is typically considered to have a lower dynamic range and specificity than RT-qPCR and RNA-seq. In addition, as circulating lncRNAs provide lower signals than do tissue lncRNAs, the increase in chip background as the expiration date approaches dramatically reduces the number of lncRNAs that can be detected.

RNA-seq is a growing approach and has the great advantage of allowing for the identification of both known and new lncRNA species. Additionally, with sample multiplexing, the cost per sample for lncRNA analysis by RNA-seq platforms can be lower than for microarray or RT-qPCR. However, standard protocols require a relatively large amount of starting material (~1 μg of RNA), which is difficult to isolate from serum or plasma. Although it is plausible that in the near future, all nucleic acid research will be performed using this methodology, it is currently expensive and requires special equipment and expert bioinformaticians; thus, it cannot be considered user or laboratory friendly.

Data normalization is another major issue regardless of the platform used for lncRNA measurement. Indeed, proper normalization with internal control(s) is critical for comparing results from different samples because it not only ensures data quality but also reduces variation introduced during sample collection, processing, and measurement. Several housekeeping genes have been successfully used as internal controls for protein-coding transcripts, though independent studies have proposed different single or sets of transcripts as candidate housekeeping markers for tissue or circulating lncRNA analysis using amplification-based methods. A global consensus has yet to be reached. Although spike-in synthetic RNAs may also be used, they cannot “remove” variation introduced prior to RNA isolation. Normalization is also a key consideration in microarray-based and sequencing-based data, and differences in normalization approaches may account for some of the inconsistencies in the results from different lncRNA-profiling studies. Therefore, the development of a standard normalization procedure is not only necessary for identifying cancer lncRNA biomarkers but also a prerequisite for using lncRNAs as diagnostic biomarkers in the clinic.

### Individual factors

In addition to the potential technical biases discussed above, other critical variables with potential deep implications for the accurate interpretation of circulating lncRNA biomarker studies are related to intrinsic inter-individual variability and the influence of disease-independent factors. When applying circulating lncRNAs as cancer biomarkers, the first source of variability that should be considered is related to the tumor itself. In fact, as lncRNA expression patterns are highly specific to individual cancers, circulating lncRNA signatures are expected to be extremely specific to different cancer types or molecular subtypes and to distinct tumor features (e.g., oncogene overexpression, hormone responsiveness). Although some inter-individual variables, such as race, gender, and age, can be easily analyzed and properly considered, there are a vast array of individual and environmental factors that are more difficult to verify and consider while evaluating circulating lncRNAs as disease biomarkers. For example, differential lncRNA expression has been attributed to polymorphisms at lncRNA chromosome loci [81]. In particular, copy-number variations (CNVs) in coding regions that are also found in lncRNA genes have been observed across all chromosomes [82]. Furthermore, CNVs that cause lncRNA deregulation have been shown to play a role in the occurrence of various diseases [83–85], and this type of polymorphism might contribute to differences in lncRNA expression among individuals as well as in the levels of specific circulating lncRNAs. Among donor-related factors, diet is a critical potential confounder in lncRNA studies [86, 87]: many of the lncRNAs contained in food could be largely indistinguishable from endogenous lncRNAs at the sequence and/or function level once they enter the circulation and could cause changes in lncRNA concentrations via homeostatic mechanisms that regulate circulating lncRNA-containing vehicles (including lipoprotein particles and exosomes). As the influence of diet and other external factors, such as physical activity, cannot be considered during case selection for circulating lncRNA studies, the measured lncRNA levels could represent a summary of individual behavior rather than of a specific disease state. Despite the lack of extensive information, intraindividual variability could affect circulating lncRNAs because lncRNA levels can vary in an individual over time. Notably, pharmacological treatments could have a profound influence on circulating lncRNA levels. For example, the effects of chemotherapy and, potentially, different types and/or treatment schedules of anticancer therapies on circulating lncRNA levels could have a large effect on these biomarkers, even though the impact of pharmacological treatments on circulating lncRNA levels suggests their potential for use as pharmacodynamic markers.

## Future perspectives and challenges of circulating lncRNAs as cancer biomarkers and potential therapeutics

Although most lncRNAs known to be dysregulated have been reported in only a single study, some have shown consistent changes in several independent studies. Systematic validation studies with randomized trials based on these “verified” lncRNAs with well-characterized and diverse patient samples may be useful. To ensure that changes in concentrations appropriately reflect pathology, a diagnostic panel should have (1) a unique lncRNA signature with high specificity and sensitivity for individual cancer types, (2) well-defined variations caused by both intrinsic and extrinsic factors, (3) standard normalization procedures with reliable internal controls, (4) well-characterized reference intervals of specific circulating lncRNAs from healthy populations, and (5) well-established concentration kinetics of specific lncRNAs in circulation. In fact, because cancerous cells represent only a small fraction of the cells in the body, most of the changes observed in specific circulating lncRNAs are a result of indirect effects of the body’s response to cancer growth. Thus, it will be difficult to develop unique circulating-lncRNA signatures for detecting specific types of cancer; instead, it may be more feasible to generate a circulating lncRNA-based pan-cancer biomarker panel to detect different cancer types.

Most studies published to date have screened for lncRNA expression in whole plasma/serum or whole urine samples. Nevertheless, there is evidence that at least a portion of the circulating lncRNA transcriptome is present in “subcompartments” of those biological samples, such as extracellular vesicles (EVs) released by cells. For instance, a proof-of-concept study demonstrated the presence of PCA3 transcripts within exosomes [88], which are nanometric lipidic vesicles secreted by cells that mediate cell-to-cell communication. A subsequent attempt to profile the genetic material enclosed within exosomes isolated from the plasma of healthy blood donors showed that lncRNAs account for 3.36 % of the total exosomal RNA content [89]. In fact, there is considerable interest in exploring the molecular content of lipid vesicles in circulation in the development of cancer-type-specific biomarkers, with the content of exosomes, specifically their miRNAs, being extensively studied as carriers of miRNA biomarkers for various diseases [90]. Different exosomal miRNAs are already well accepted as biomarkers for the diagnosis and prognosis of different types of cancer [91], and lncRNAs are being pursued for similar goals. Although the concept is reasonable, few studies to date have utilized purified lipid vesicles for biomarker discovery [49, 92, 93], likely because of the difficulty in rapidly and reliably obtaining vesicles from a large number of samples. Although several commercial kits are available for vesicle isolation, most employ polymer-based precipitation of particles of certain sizes; therefore, the vesicles obtained using these kits may contain other molecular complexes in circulation. Traditional ultracentrifugation-based methods provide cleaner vesicle populations but are time consuming and require larger sample volumes, making this approach impractical when considering a large number of samples in biomarker-discovery projects. Accordingly, a better isolation method is needed to facilitate the use of lipid vesicles in biomarker discovery. With increased effort in characterizing the content of lipid vesicles and understanding the cellular processes of selecting and packaging molecules into lipid vesicles, it might be possible to identify and use cancer-derived vesicles in the circulation to detect specific cancer types and stages.

**Fig. 1:**
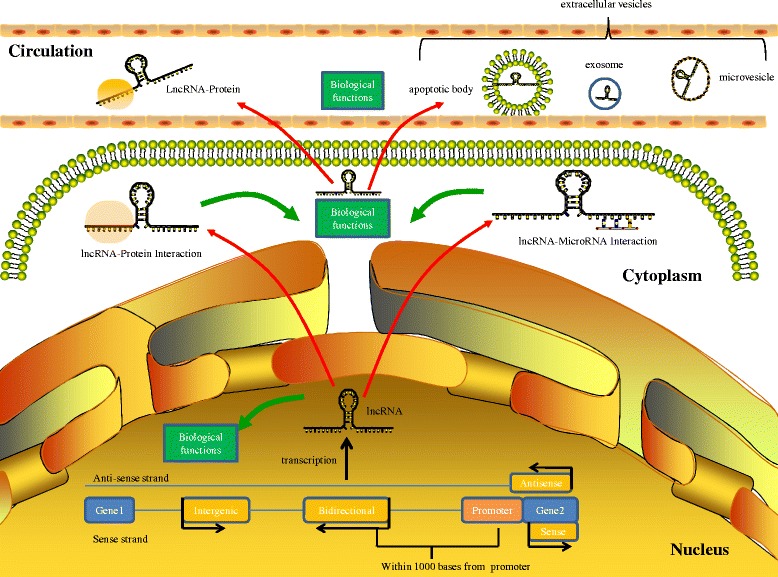
Biogenesis and function of lncRNAs

**Fig. 2:**
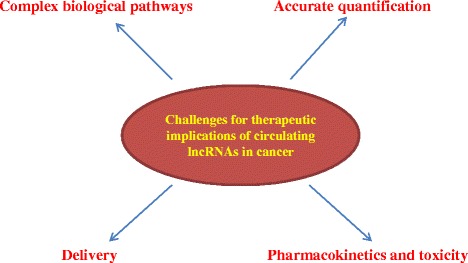
Challenges for therapeutic implications of circulating lncRNAs in cancer

Given that EVs play an important role in the regulation of cell-cell communication, EV-derived lncRNA may be transferred from donor cells to receptor cells, functioning in target cells in diverse biological processes, including the regulation of tumorigenesis. A study by Kogure et al. showed that the EV-mediated transfer of lncRNA-TUC339 from tumor cells can promote the growth and spread of HCC [94]. Takahashi and colleagues also observed that cell behavior can be modulated by linc-VLDLR within EVs [95]. Although information on circulating lncRNA in EVs has only been recently reported and the precise role of circulating lncRNAs in cancer development via cell-cell communication requires further investigation, these findings support the hypothesis that lncRNAs within EVs have an important role in cancer-non-cancer-cell crosstalk within the tumor microenvironment. These lncRNAs may thus be useful as therapeutic tools for treating cancers in diverse pathological conditions (Fig. 1). However, several problems have been encountered during the clinical development of EV lncRNA-based therapeutics for tumors (Fig. 2), including their uncertain functions in the complex networks of biological pathways, accurate quantification of EV-derived lncRNAs, the delivery of circulating lncRNA antagonists or mimics and the clinical pharmacokinetics and toxicity of lncRNAs.

## Conclusions

The discovery of circulating lncRNAs has enabled a new understanding of the basic mechanisms of oncogenesis and opened up exciting prospects for diagnostics and prognostics. Although circulating lncRNAs still represent a new field with much to be explored, the goal is to apply these lncRNAs for diagnosing and treating cancer when we know more about their origin and function. However, as discussed in this review, several challenges must be overcome to further develop circulating lncRNA-based biomarkers for clinical applications. A comprehensive understanding of the factors that may affect lncRNA measurement will help in establishing a commonly acceptable procedure for sample collection, storage and processing as well as lncRNA measurement. Indeed, the development and application of SOPs related to circulating lncRNA analysis is imperative for successfully translating lncRNA signatures into clinically meaningful tests. To minimize interlaboratory and inter-user differences that could have major impacts on lncRNA results, standardized and consistent methods must be applied at many levels, from whole-blood collection to plasma/serum preparation, handling, and banking to RNA extraction and lncRNA quantification. Additionally, the establishment of reference sample sets for both cancer and normal controls is important for generating a consensus and facilitating validation. Once consensus procedures have been defined and included for profiling circulating lncRNA, it will be possible to interpret and compare different study results and potentially identify lncRNAs acting as novel specific and sensitive cancer biomarkers. More importantly, an understanding of the fundamental biology involved in intra- and intercellular lncRNA trafficking is critical for interpreting the fundamental biology and pathology reflected in changes in the concentrations of specific circulating lncRNAs. With a better understanding of the molecular properties of circulating lncRNA, it may be possible to offer a more sensitive and accurate biomarker for early cancer detection or to improve the performance of existing clinical biomarkers.
